# Neuropharmacological potential of honokiol and its derivatives from Chinese herb *Magnolia* species: understandings from therapeutic viewpoint

**DOI:** 10.1186/s13020-023-00846-1

**Published:** 2023-11-24

**Authors:** Md. Faysal, Jishan Khan, Mehrukh Zehravi, Nikhil Nath, Laliteshwar Pratap Singh, Saloni Kakkar, Rajashekar Perusomula, Pathan Amanulla Khan, Firzan Nainu, Mohammed Asiri, Sharuk L. Khan, Rajib Das, Talha Bin Emran, Polrat Wilairatana

**Affiliations:** 1 Department of Pharmacy, Faculty of Allied Health Sciences, Daffodil International University, Dhaka, 1207 Bangladesh; 2 Department of Pharmacy, International Islamic University Chittagong, Kumira, Chittagong, 4318 Bangladesh; 3 Department of Clinical Pharmacy, College of Dentistry & Pharmacy, Buraydah Private Colleges, 51418 Buraydah, Saudi Arabia; 4 Department of Pharmaceutical Chemistry, Narayan Institute of Pharmacy, Gopal Narayan Singh University, Jamuhar, Sasaram, (Rohtas), Bihar 821305 India; 5 Department of Pharmaceutical Sciences, Maharshi Dayanand University, Rohtak, Haryana 124001 India; 6 Cognitive Science Research Initiative Lab, Vishnu Institute of Pharmaceutical Education & Research, Narsapur, India; 7 Department of Pharmacy Practice, Anwar Ul Uloom College of Pharmacy, New Mallepally, Hyderabad, India; 8 Department of Pharmacy, Faculty of Pharmacy, Hasanuddin University, Makassar, 90245 Indonesia; 9 Department of Clinical Laboratory Sciences, College of Applied Medical Sciences, King Khalid University, Abha, Saudi Arabia; 10 Department of Pharmaceutical Chemistry, N.B.S. Institute of Pharmacy, Ausa, Maharashtra 413520 India; 11 Department of Pharmacy, Faculty of Pharmacy, University of Dhaka, Dhaka, 1000 Bangladesh; 12 Department of Pathology and Laboratory Medicine, Warren Alpert Medical School & Legorreta Cancer Center, Brown University, Providence, RI 02912 USA; 13 Department of Clinical Tropical Medicine, Faculty of Tropical Medicine, Mahidol University, Bangkok, 10400 Thailand

**Keywords:** Honokiol, Chinese Herb, *Magnolia* species, Neurological diseases, Alzheimer’s disease, Parkinson’s disease, Ischemia, Anxiety, Depression

## Abstract

Honokiol is a neolignan biphenol found in aerial parts of the *Magnolia *plant species. The *Magnolia* plant species traditionally belong to China and have been used for centuries to treat many pathological conditions. Honokiol mitigates the severity of several pathological conditions and has the potential to work as an anti-inflammatory, anti-angiogenic, anticancer, antioxidant, and neurotherapeutic agent. It has a long history of being employed in the healthcare practices of Southeast Asia, but in recent years, a greater scope of research has been conducted on it. Plenty of experimental evidence suggests it could be beneficial as a neuroprotective bioactive molecule. Honokiol has several pharmacological effects, leading to its exploration as a potential therapy for neurological diseases (NDs), including Alzheimer's disease (AD), Parkinson’s disease (PD), cerebral ischemia, anxiety, depression, spinal cord injury, and so on. So, based on the previous experimentation reports, our goal is to discuss the neuroprotective properties of honokiol. Besides, honokiol derivatives have been highlighted recently as possible therapeutic options for NDs. So, this review focuses on honokiol's neurotherapeutic actions and toxicological profile to determine their safety and potential use in neurotherapeutics.

## Introduction

Neurological diseases (NDs) affects the nervous system, causing symptoms due to body composition or metabolism issues. These symptoms can result from various health issues [1, 2]. Common symptoms of non-infectious brain diseases include muscle paralysis, slurred speech, seizures, and hand numbness. These disorders can lead to NDs such as epilepsy, Alzheimer’s disease (AD), and Parkinson’s disease (PD), causing high mortality rates and cognitive impairment [3]. Aging populations are expected to increase the number of people with disabilities. Mainly, these diseases are more common in the elderly. Age-related conditions are gradually more prevalent, placing massive economic and social pressure on families and communities [4]. Patients with PD and Huntington’s disease (HD) have dopaminergic and spiny striatal neurons that control their movements. AD is characterized by a drop in the brain’s total volume. A condition that affects the nervous system can damage the brain's neuron count. However, to this point, there is still a lack of effective therapeutics for devastating brain disorders. In this regard, the attempt to discover novel, effective molecules should be a crucial research goal [5, 6].

Honokiol (3',5-di-(2-propenyl)-1,1'-biphenyl-2,4'-diol) is a phenylpropanoid molecule that belongs to the category of neolignans. Neolignans belong to a group of plant-based phytochemicals similar to lignans. The shikimic acid pathway can be followed to proceed with the biosynthetic procedures. It contains a para-allyl-phenol and an ortho-, ortho-allyl-phenol linked through a para-C–C coupling [7, 8]. In Asian regions, honokiol is a commonly used ingredient in homeopathic medicine. The potential source of honokiol may be *Magnolia Officinalis,* which is generally grown in China. Raw materials from Magnolia tree bark, seed cones, and leaves are necessary to extract the lignan honokiol in various plant parts successfully. Honokiol is often used as an effective therapeutics for several medical conditions [9]. The anxiolytic, analgesic, depressive, antithrombotic, antibacterial, antispasmodic, anti-tumorigenic, and neuroprotective effects of honokiol are well-known [10, 11].

Honokiol is a polyphenolic compound with a small molecular size. Its high lipophilicity enables it to cross the blood brain barrier (BBB) and blood-cerebrospinal fluid (CSF) barrier easily. Currently, many research reports indicate that it exhibits significant neuroprotective properties in several animal models of central nervous system (CNS) disorders, such as spinal cord injury, epilepsy, cerebrovascular injury, anxiety, and cognitive disorders. This is attributable to its ability to prevent oxidative damage and neural excitotoxicity, mitigate neuroinflammation, and regulate the functioning of mitochondria [3, 10, 12, 13]. As per a study, honokiol effectively treats PD neuronal and motor impairments by reducing oxidative damage and inflammation, improving overall health [14].

Furthermore, honokiol has been demonstrated to benefit cognitive functioning in APP/PS1 by mitigating mitochondrial damage [13, 15]. The administration of honokiol was found to mitigate cerebral edema and neurobehavioral impairments following subarachnoid hemorrhage by enhancing mitochondrial fusion. This mechanism helped to maintain the structure of mitochondria, safeguard their functionality, and support the survival of neural cells. Honokiol has also been submitted for patent (200310121303.0) as a therapeutic option for ischemic stroke, and clinical trials are expected to commence in China soon [13].

It is already established that honokiol can cross the BBB, and many researchers have argued that honokiol could be a successful therapeutics for various NDs [16, 17]. The neuroprotective effects of honokiol and its derivatives have recently been under increased scrutiny. Honokiol's derivatives were focused on their neurotherapeutic effects and toxicological profile to determine if they could be less hazardous for use.

## Honokiol derivatives

Several neuroprotective honokiol derivatives include acetate, glycerol, succinic acid, glucose, glycine, and mannose derivatives. These derivatives are used to develop new compounds with enhanced solubility properties. According to a study, 2- and 4'-OH groups acetylation of honokiol was accomplished by incubating the molecule in acetic anhydride in pyridine for 18 h at 25 °C. This causes the honokiol molecule to become acetylated [18, 19]. As a result, a chemical by-product, honokiol-acetate, is produced. Honokiol-succinic acid was formed by the reaction between honokiol and maleic anhydride (3,4-dihydrofuran-2,5-dion) in pyridine over honokiol-acetate at room temperature (50 °C) [20]. The honokiol-glycine derivative was synthesized by reacting honokiol with glycerol in pyridine for eight hours at 25 °C. Following the reaction of glycine with di-tertbutyl dicarbonate (di-BOC), a synthetic Boc-Gly honokiol called 1,3-diaza-1,3-dicyclohexylpropa-1,2-diene (DCC) is then reacted with triethylamine. Boc-Gly honokiol and 4-dimethyl aminopyridine (DMAP) at 40 °C for six hours. This peptide goes by no specific name. In the next step, a di-Boc on di-Boc-Gly-honokiol was removed by bubbling the EtOAc solution with dry HCl gas at room temperature for four hours. This step constituted the second part of the process. This phase is completed after the second phase [19, 21]. Besides, the processes of glycosylation and deacetylation lead to the formation of honokiol-glycine and honokiol mannose molecules. In the first step of the process, diethyl ether was used to incorporate boron trifluoride into honokiol-glucose, honokiol mannose glucose pentaacetate, and mannose pentaacetate (BF3-Et2O). This results in synthetic honokiol-β-D-tetra acetyl glucopyranoside and honokiol-β-D-tetra acetyl mannopyranoside. In the second step, sodium methoxide cleaves synthetic honokiol-β-D-tetra acetyl glucopyranoside and honokiol-β-D-tetra acetyl. Following this step, the molecule was deprotonated by passage through an Amberlite IR120 (H^+^) ion exchange column, yielding honokiol. Once this part of the process was complete, the structures of the newly synthesized honokiol-derivatives were determined based on ^1^H-and ^13^C-NMR spectrum data collected from those compounds [8, 19, 22, 23].

Besides, many experimental studies reported enormous honokiol derivatives with better therapeutic efficacy [24–28]. For example, experimental investigations were performed on the bark portion of *M. officinalis* and a total of four novel derivatives of honokiol were successfully isolated, namely (1) 8′,9′-dihydroxymagnaldehyde E, (2) 8′, 9′-dihydroxy Honokiol, (3) erythro-7-O-methylhonokitriol, and (4) threo-7-O-methylhonokitriol. Detailed 1D and 2D NMR investigations and HR-MS analysis served as a base for developing their molecular structures [29].

In another experiment, 4-o-methyl honokiol has gone through synthetic modifications. The experimental findings have been attributed to synthesizing of 31 neolignan honokiol analogs. Xenopus oocytes were used as a model system to investigate how the synthetic analogs work on the GABA_A_ receptors. Honokiol increased chloride currents across receptors expressing GABA_A_, with EC50 concentrations that ranged from 23.4 μM to 59.6 μM in 7 distinct subunit compositions. The researchers have developed GABAA receptor modulators that exhibit remarkable subtype-selectivity by selectively modifying crucial functions of honokiol, such as its phenolic group, side chains, polarity, and ring positions for substitutions. Compounds composed of amino groups are a newly identified class of GABA_A_ receptor modifiers that exhibit high potency and efficacy. Within this class, several potential lead candidates show promising benefits. The majority of synthetic analogs have yet to be documented. The efficacy of compounds 26 and 31 was approximately 10 times greater than honokiol’s effect [30].

## Structural-activity relationship (SAR)

**Fig. 1:**
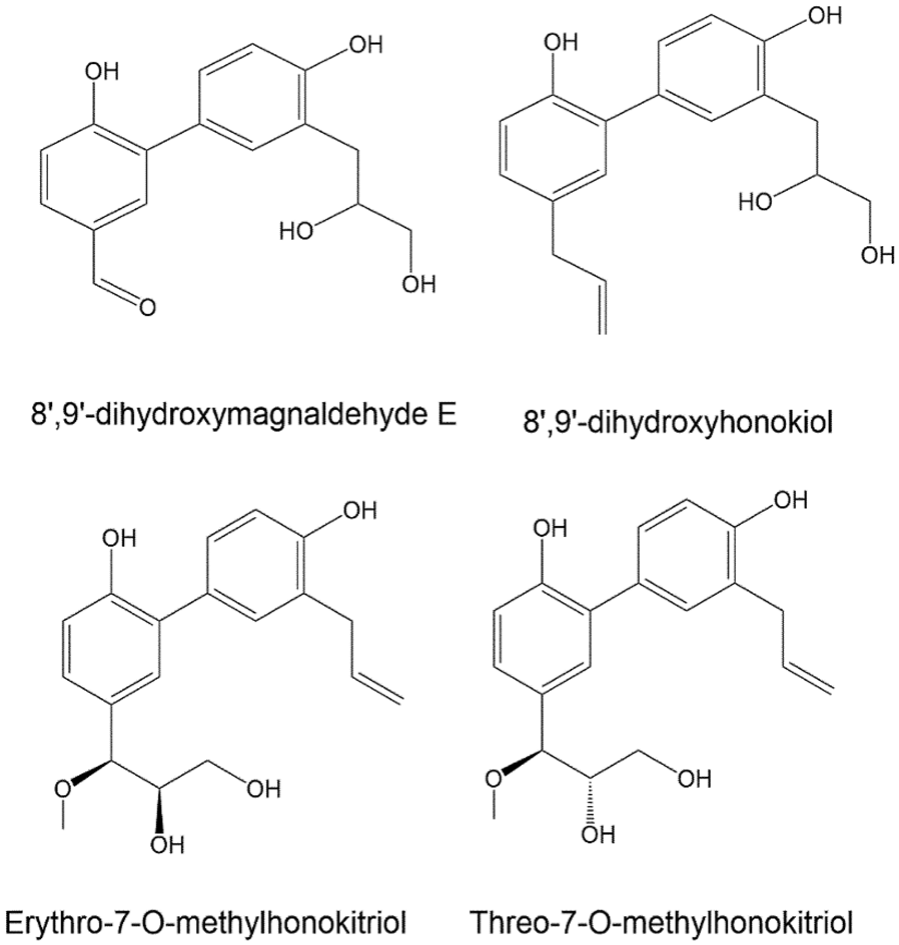
Four novel derivatives of honokiol by Zhang et al. [29]

**Fig. 2:**
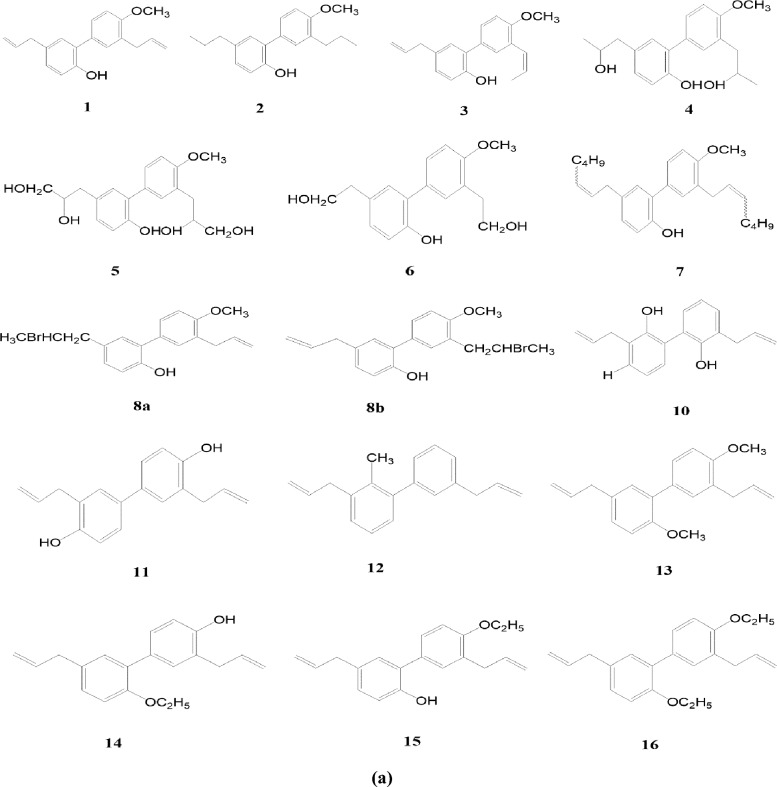

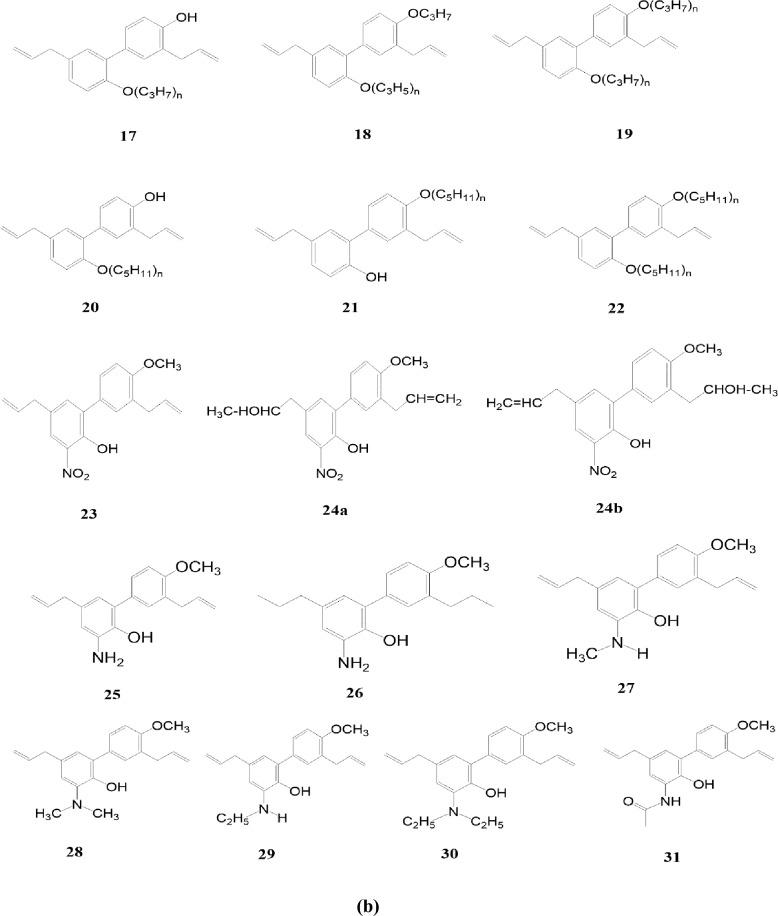
**a** 31 neolignan honokiol analogues of 4-o-methylhonokiol by Talarek et al. [30]. **b** 31 neolignan honokiol analogues of 4-o-methylhonokiol by Talarek et al. [30]

Honokiol, a naturally occurring bioactive substance in magnolia tree bark, has potential therapeutic benefits such as anti-inflammatory, antioxidant, neuroprotective, and anticancer actions. Researchers have investigated its derivatives to improve their pharmacological characteristics and maximize their action. The structural-activity relationship (SAR) of honokiol and its derivatives consists of numerous hydroxyl (-OH) groups that support its antioxidant and free radical scavenging properties [31, 32]. The absorption and distribution of a molecule within a cell can be influenced by its lipophilicity, which is the degree to which it can dissolve in lipid-based environments. Changes to honokiol derivatives’ alkyl or aryl groups can affect their lipophilicity and bioavailability [33]. The biological effects of honokiols can alter when functional groups such as methyl, ethyl, halogen, or amino groups are added to the compound’s structure. For example, adding electron-donating groups may improve its antioxidant properties [31]. The biphenolic structure of honokiol is essential for its biological function, and interactions with biological targets and receptors can be altered by changing the rings or adding fused ring systems [34]. These results allow for innovative novel honokiol compounds with enhanced biological activity. For example, honokiol can improve its solubility and bioavailability by changing its propyl group to a more hydrophobic one [31]. Research on honokiol and its derivatives has shown its versatility in targeting various targets. They can modulate GABA_A_ receptors, which control anxiety, pain, and seizures, and block NMDA receptors, which control pain, neurodegeneration, learning, and memory [11]. There are several potential therapeutic uses for honokiol and its derivatives, but further study is needed to fully understand their mode of action and develop secure and efficient treatments (Figs. 1, 2).

**Fig. 3:**
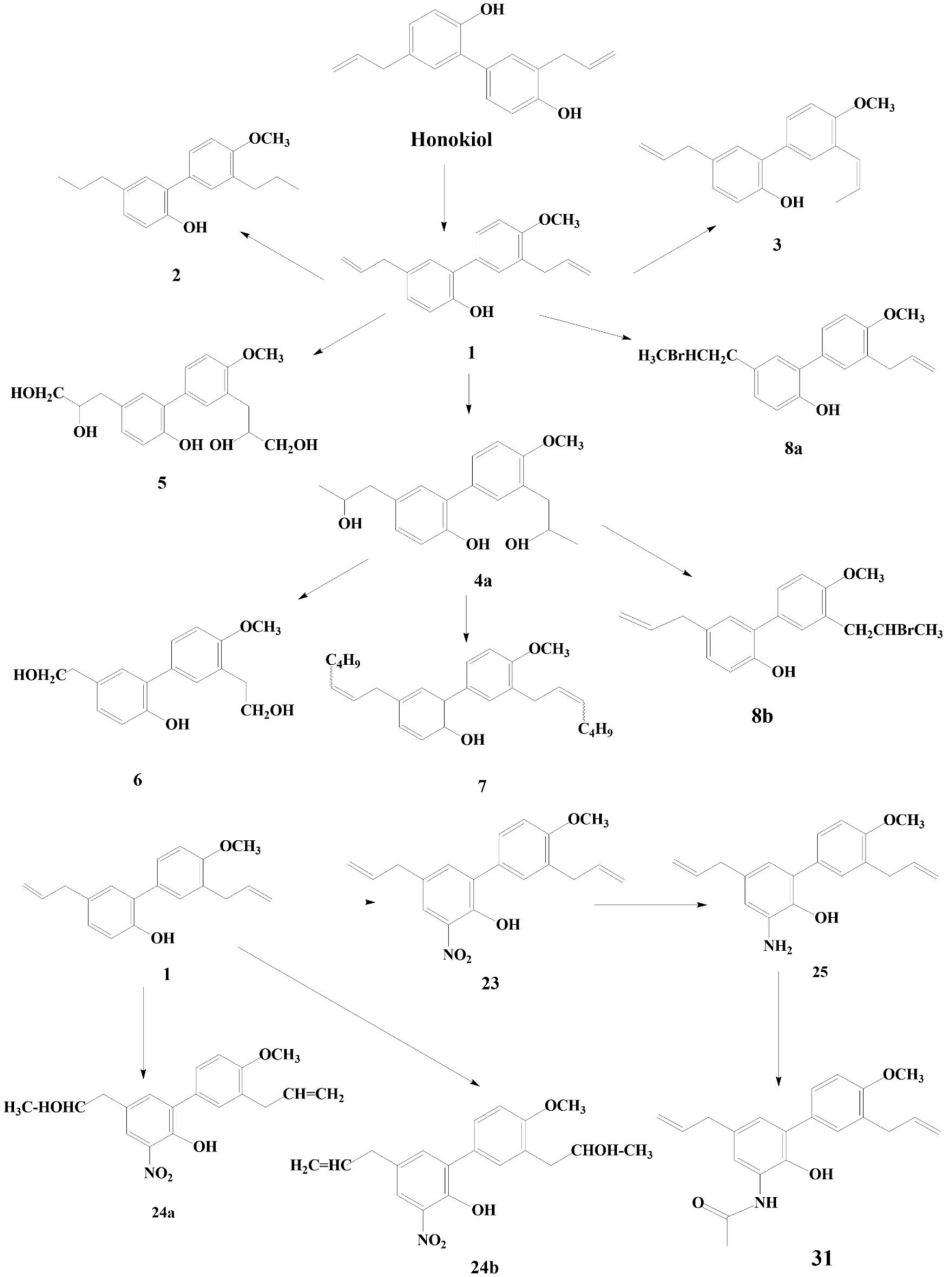
Structural-activity relationship of Honokiol

A study on the effects of honokiol and its derivatives on GABA_A_ receptors revealed that these compounds enhanced chloride currents [30]. The potency of these compounds varied depending on the receptor subtype, with honokiol being most potent on α3β2 receptors and 3'-acetylamino-4′-O-methylhonokiol being most potent on α2β2 receptors. Functional groups like methoxy and amino groups could improve the solubility and bioavailability of honokiol derivatives, but these modifications could also reduce their biological activity. The study focused on the SAR in terms of potency (EC_50_), which indicates receptor affinity. The initial study focused on aliphatic side chain modifications deriving from 4'-O-methyl honokiol (1), producing derivatives 2-8b. The study found that tetrahydro methyl-honokiol 2 was twice as active as 1, and isomerization of the double bond in ring B's side chain slightly reduced the EC_50_. Hydrobromination in one of the side chains significantly increased the potency of IGABA when the double bond of ring A's side chain was modified. Incorporating a nitro group as a solid electron-drawing entity into ring A resulted in marginally increased biological activity compared to honokiol. Compounds 24a and 24b displayed improved potency due to an unexpected acetylamino group introduction to 1’s side chain (Fig. 3). However, efficacy lagged behind honokiol. A reduction of 23 resulted in 3-amino-4'-o-methylhonokiol, which exhibited a twofold IGABA potentiation (2179%) within the dataset. The acetylated amine 3-n-acetylamino-4'-o-methylhonokiol emerged as a stable, long-lasting derivative with high potency and efficiency (2601%) [30]. This study provides valuable insights into the structure–activity relationship of honokiol and derivatives. The findings of this study could be used to design new compounds with improved biological activity.

## Neurotherapeutic properties of honokiol

### Alzheimer’s disease (AD)

AD is a progressive neurological disorder and one of the common causes of dementia in people worldwide [3]. AD can be diagnosed based on beta-amyloid (Aβ) aggregates and senile plaques in the cerebral cortex and hippocampus. Abnormal production and accumulation lead to irreversible neuronal cell death [35]. A primary goal of AD research in the modern era is to improve the cellular breakdown system to eliminate Aβ and inflammation as promptly and efficiently as possible. PPAR-γ, known as peroxisome proliferator-activated receptor-β, is the most prominent transcription factor in the ligand-activated nuclear receptor superfamily [36, 37]. There is mounting evidence that PPAR-γ modulates microglia in healthy and diseased states. In addition, PPAR-γ is identified as a therapeutic target for AD. First, PPAR-γ activation increases the expression of its target genes, LXR, CD36, and SRA, and this increase is dose-dependent [38]. By increasing CD36 expression, microglia acquire a more remarkable ability to phagocytose Aβ. Activation of genes such as ABCA1 and ApoE by the transcription factor LXR is a means by which Aβ can be efficiently removed from microglia. An additional effect of PPAR-γ activation is that it interacts with other transcription factors, reducing the amount of DNA binding activity that nuclear factor kappa-B (NF-kB) can [39]. The action of E3 ligase, which PPAR-β regulates, ultimately leads to the degradation of NF-kB/p65. Aβ ubiquitous transcription factor known as NF-kB is responsible for the transcription of various cytokines that promote inflammation. Oxidative stress (OS) is caused by the inappropriate folding of antibodies, which are essential for mitochondrial membrane potential. Sirtuin-3 (SIRT3) degradation activity helps ensure this anomaly does not occur [39, 40]. It has been demonstrated that an essential function of SIRT3 is to protect neurons against excitotoxicity. In the past, the only way to increase SIRT3 levels was to adhere to a strict calorie restriction plan and engage in endurance activity [40, 41]. Plenty of experiments reported that honokiol could be a good choice.

Honokiol has been identified as a potent activator of SIRT3, demonstrating antioxidant properties and enhancing the functioning of mitochondria across multiple experimental designs. Recent evidence indicates that SIRT3 is crucial in advancing various metabolic processes and neurodegenerative ailments. People with AD have an accumulation of Aβ, which is the prelude of extracellular senile plaques, in their brains. This accumulation is associated with a progression of cognitive dysfunction and the loss of neural cells. Aβ is produced via a series of proteolytic cleavages of Aβ precursor protein (APP), initially by β-secretase followed by γ-secretase. Pharmaceutical agents that regulate this particular pathway are widely considered to be among the most promising approaches to the medical management of AD. Ramesh et al. [42] found that honokiol, a compound with the APP and Presenilin PS1 mutation, affected Chinese Hamster Ovary (CHO) cells. They found that honokiol stimulated the expression of SIRT3, reduced reactive oxygen species (ROS) generation, improved antioxidant properties, and increased mitochondrial functionality. This led to a reduction in Aβ and sAPPβ levels.

**Fig. 4:**
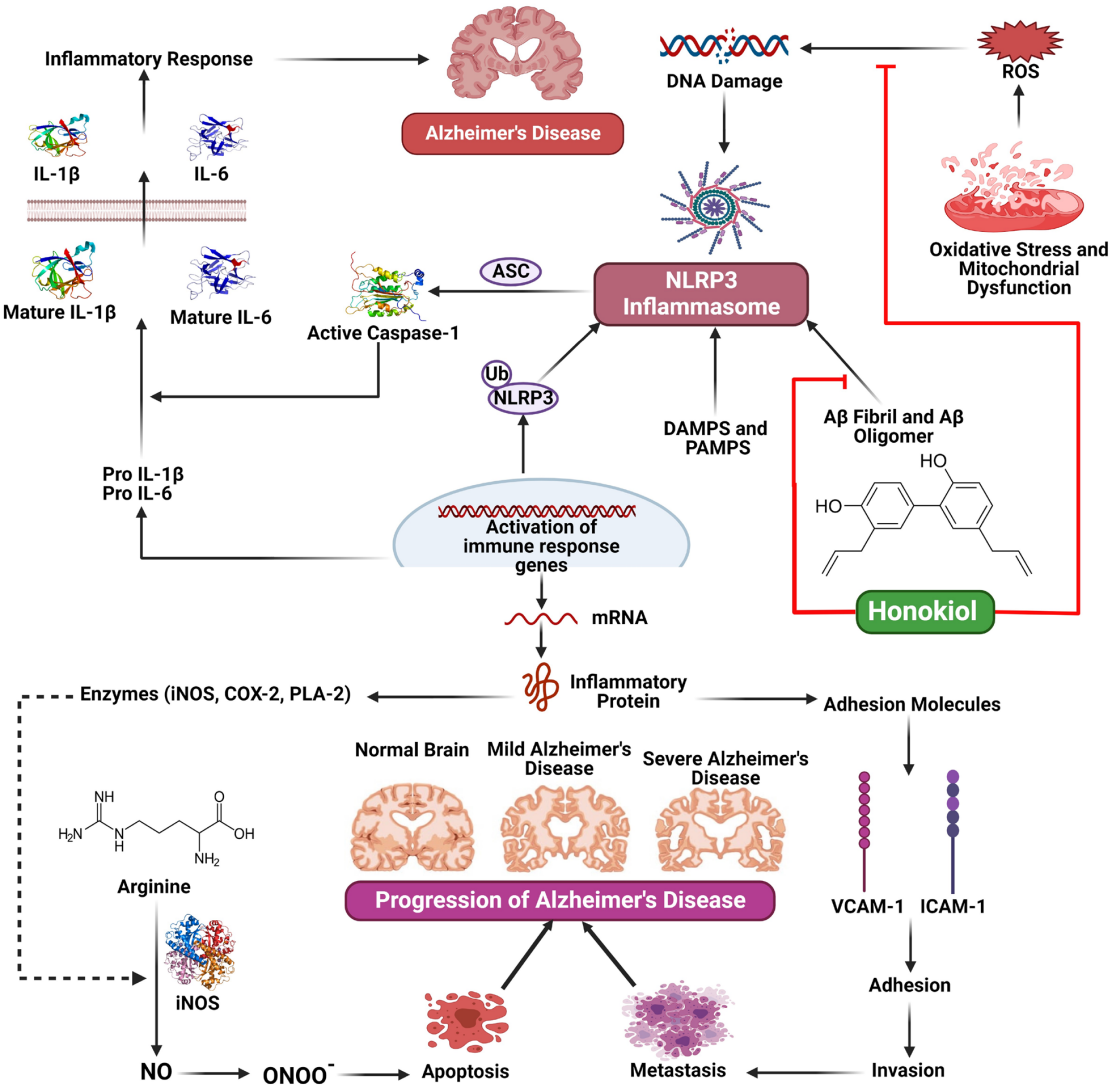
Mechanistic illustration of the therapeutic applications of honokiol in Alzheimer’s disease, and Parkinson’s disease

The study found that honokiol did not affect APP protein concentrations or α-secretase function. Instead, it enhanced AMPK, CREB, and PGC-1α expression, inhibiting β-secretase activity and decreasing Aβ levels. This suggests that honokiol has SIRT3 activator properties, enhancing antioxidant function and controlling mitochondrial energy. This makes it a promising candidate for AD drug development [42]. According to the findings of Wang and colleagues, giving honokiol intraperitoneally to APPswe/PS1dE9 transgenic mice at a dose of 20 mg/kg/day for six weeks significantly restored their spatial memory deficiencies. The study found that honokiol significantly reduced the production of Aβ and the deposition of senile plaques. This was achieved through the downregulation of β-site precursor protein for amyloid cleavage enzyme 1 and the enhancement of Aβ phagocytosis by microglia. The administration of honokiol decreased the activation of glial cells and reduced the synthesis of pro-inflammatory cytokines, including tumor necrosis factor alpha (TNF-α), IL-1β, and IL-6 **(**Fig. 4**)**. The transcriptional functions and protein concentrations of PPARγ were enhanced by honokiol. The advantageous effects associated with honokiol on pathological alterations, encompassing biochemistry and cognitive function, were found to be impeded by GW9662, a particular PPARγ inhibitor. The results of this study indicate that honokiol has the potential to act as a natural agonist for PPARγ**,** thereby reducing both neuroinflammation and Aβ generation. Thus, honokiol exhibits promise as a prospective therapeutic strategy for treating AD [43].

According to Lee et al. [44]’s findings, administering a 10 mg/kg dose of an ethanol extract of *M. officinalis* reduced memory loss in an LPS-induced model of AD in mice using the water maze test [44]. A further investigation was conducted to assess the impact of honokiol on the impairment of memory and learning induced by AβO in mice. The AO intrahippocampal injection was used for developing a mouse model of AD. A Morris water maze (MWM) test measured the subject's cognitive ability. The administration of honokiol resulted in a significant rise in the duration of time spent by the mice with AD in the designated zone during the MWM test.

Furthermore, it was observed that honokiol exhibited a dose-dependent reduction in hippocampal neural apoptosis, ROS generation, and a decline in the membrane potential of mitochondria caused by AβO. In addition, the stimulation of NF-κB caused by AβO was suppressed by honokiol, along with the increased expression of APP and β-secretase [45]. A study investigated the potential benefits of honokiol against mitochondrial abnormalities caused by AβOs in neuronal cells and its impact on cognitive abilities in transgenic AD mice. The findings showed that honokiol increased mitochondrial SIRT3 expression, increasing ATP generation and reducing ROS production. This suggests that honokiol may be therapeutically helpful in postponing the onset of AD symptoms by reducing mitochondrial dysfunction and enhancing SIRT3 hyperactivation [46]. Subsequent investigations have suggested that the neurotherapeutic properties of the molecule, as mentioned above, may be attributed to the mitigation of ROS generation, as well as the curtailment of intracellular calcium upregulation and the hindrance of caspase-3 enzymatic activity. This study's findings offer a scientific justification for the therapeutic application of honokiol in managing AD [1].

According to Matsui et al., [47] honokiol was administered to SAMP8 mice at an oral dose of 0.1 and 1 mg/kg daily for 14 days when the mice were 2 months old. At 2 months of age, the forebrain of SAMP8 mice exhibited reduced activity of Akt, which is a constituent of the prosurvival pathway. The administration of honokiol at a dose of 1 mg/kg for 14 days increased Akt phosphorylation levels in the forebrain region at the age of 2 months. The findings indicate that honokiol may protect against cognitive decline associated with aging by maintaining the integrity of cholinergic neurons in the forebrain. The molecules mentioned above exhibit promising potential as therapeutics for NDs [47].

A study aimed to determine the potential of honokiol in reversing memory and learning deficits caused by scopolamine in mice. The researchers administered 10 and 20 mg/kg of honokiol intraperitoneally for 21 days, substantially enhancing memory and spatial learning function. The administration also reduced the synthesis of prostaglandin E2 (PGE2) and COX-2 mRNA expression in the cerebral cortex of mice administered scopolamine. The mechanistic assessments showed that honokiol significantly reset alterations in phosphorylated Akt and ERK1/2 levels in the cerebral cortex of mice administered scopolamine. The findings support the notion that honokiol has the potential to enhance cognitive function and alleviate memory deficits caused by scopolamine in mice. This protective effect is likely due to suppressing AChE activity and mitigating neuroinflammation in the SCOP-administered mice [48].

### Parkinson’s disease (PD)

Neuroinflammation is a complex process that plays a role in various complex conditions affecting the brain, including ischemia injury and neurodegenerative diseases such as multiple sclerosis and PD [49]. Atrophy of projection fibers from the substantia nigra pars compacta to the striatum results from the progressive death of substantia nigra pars compacta dopaminergic (DA) neurons, the most frequent movement state in adults. Activation of glial cells, such as astrocytes and microglia, is a pivotal biological process in neuroinflammation. Astrocytes and microglia undergo substantial structural changes during inflammation, followed by increased production of essential immunomodulatory cytokines [50, 51]. Consequently, astrocytes and microglia, under conditions of inflammatory stress, can produce several cytokines that promote inflammation. These cytokines include IL-6, TNF-α, and IL-1β [52]. Other transcription factors, such as the well-known regulator NF-kB and Krüppel-like factor 4 (KLF4), are involved in similar regulatory pathways that regulate the expression of pro and anti-inflammatory cytokines. Indeed, in addition to its actions in maintaining stem cells, KLF4 was associated with the overexpression of IL-10 and iNOS in macrophages and IL-6 in dendritic cells [53]. Endothelial cells and microglia-like cells belonging to the immune system may benefit from the role that KLF4 plays in regulating pro-inflammatory mediators [54].

**Fig. 5:**
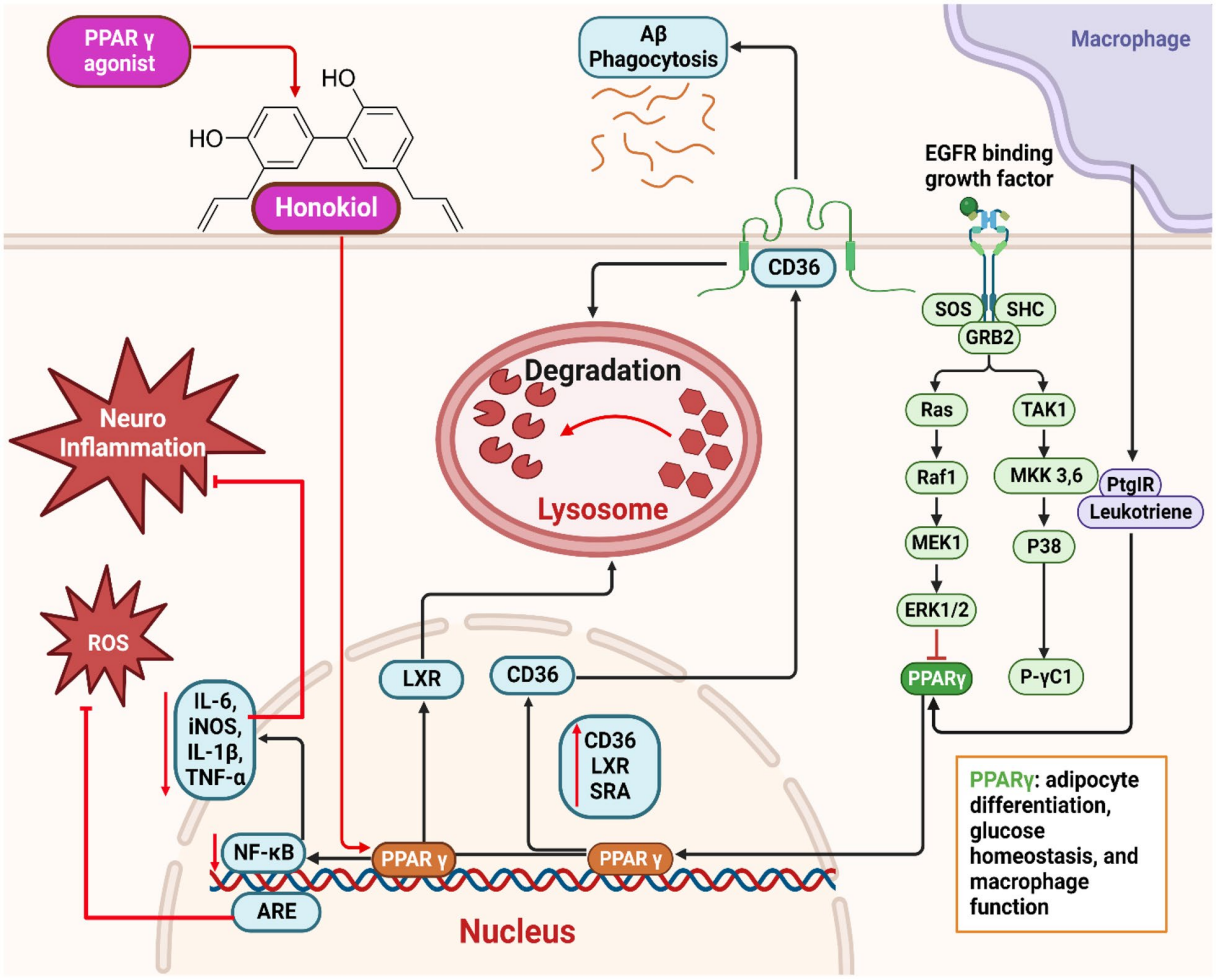
Mechanistic illustration of the therapeutic applications of honokiol in neuroinflammation. Honokiol has the potential to bind and activate peroxisome proliferator-activated receptor gamma (PPAR-γ), subsequently leading to the induction of liver X receptor (LXR), scavenger receptor A (SRA), and cluster of differentiation 36 (CD36) expression. These molecular mechanisms are responsible for facilitating the efficient lysosomal clearance of amyloid-beta (Aβ) in microglial cells. Simultaneously, Honokiol demonstrated inhibitory effects on NF-κB activity and mRNA expression of inflammatory cytokines. Furthermore, it has the capability to stimulate the Nrf2-ARE pathway and mitigate the generation of reactive oxygen species (ROS)

Many PPARγ agonists have been shown to provide neuroprotective benefits in both in vitro and in vivo models. Dozens of different animal models of PD have revealed that PPARγ activation reduces levels of OS and inflammation, improves mitochondrial function, reduces glial activation, slows the rate of nigral dopaminergic neuron loss, and increases striatal dopamine levels (Fig. 5) [55, 56]. These outcomes conclude that PPARγ may be a therapeutically productive target for treating PD. So, therapeutic interventions targeting PPARγ in people with PD may effectively reduce the severity of disease symptoms and halt the long process of neurodegeneration [57, 58].

*M. Officinalis* contains the major bioactive polyphenols honokiol and magnolol, often used in various neurological conditions, including anxiety and nervous tension. In rat models of PD, their two main compounds, magnolol, and honokiol, reduced the extent of nerve damage and behavioral problems. In in vivo and in vitro models of PD, the neuroprotective benefits of honokiol and magnolol can be attributed to the reduction of OS, glial activation, excitotoxicity, and neuronal inflammation (Fig. 5) [31]. Each of these factors plays a role in neurotoxin-induced neurotoxicity and motor impairment. Also, it has been found that honokiol was more effective than magnolol in protecting neurons from damage caused by neurotoxins and oxidants [31, 59]. In addition, honokiol is a retinoid X receptor (RXR) dimer activator and a PPARγ gamma agonist. This study aimed to demonstrate the therapeutic potential of honokiol for neural regeneration activity in PD and hemiparkinsonian rats by suppressing PPARγ activation in rat unilateral striatum using 6-hydroxydopamine injection (6-OHDA) [60].

Honokiol has been shown to have neuroprotective effects, particularly in the context of dopaminergic neuronal degeneration and motor impairments in mice following unilateral striatal injection of 6-OHDA. A recent study found that honokiol reduced the effects of 6-OHDA changes and improved contralateral rotation caused by apomorphine. Additionally, administration of honokiol in the after-treatment phase reduced impaired motor functioning and improved the loss of tyrosine hydroxylase-immunoreactive neurons. The administration of honokiol also reversed the increased expression of inducible nitric oxide synthase (iNOS) in the nigrostriatal region and reduced neuronal nitric oxide synthase (NOS) induced by 6-OHDA. These findings suggest that honokiol has protective and curative functions against motor deficits and dopamine-responsive progressive impairment in mice with 6-OHDA-induced lesions. This may be due to the modulation of NOS signaling. Honokiol can potentially serve as a therapeutic agent for treating neurological symptoms and neuronal degeneration in PD [14]. A subsequent investigation by the same researchers revealed that the inhibitory impact of PPARγ antagonist GW9662 prevented reversing the benefits of honokiol on behavioral problems and striatal PPARγ expression. Notably, the administration of subchronic honokiol resulted in an extension of the life expectancy of mice exhibiting hemiparkinsonian symptoms. The results of this study indicate that honokiol possesses beneficial properties that can improve motor function and prevent dopaminergic injury by modulating PPARγ signaling. Thus, the triggering of PPARγ by Honokiol presents a promising avenue for managing motor manifestations and neurodegeneration in PD, making it an appealing treatment candidate [61]. The neuroprotective potential of Honokiol against PD was investigated by Xu et al. in a model in mice. Sixty male C57BL/6 mice were randomly assigned to five distinct groups: the control group, amantadine group, mitochondrial toxin 1-methyl-4-phenyl-1,2,3,6-tetrahydropyridine (MPTP) model group, and honokiol groups A and B. The study employed a spontaneous movement test to assess the activity frequency of individual mice over 5 min. Additionally, HPLC-electrochemistry was utilized to identify any alterations in dopamine levels in the striatum of the mice. The study's findings indicated that the frequency of spontaneous movement exhibited by mice in the MPTP model group was comparatively lower than that of the control group over 5 min [62].

The compound honokiol has been observed to decrease the inflammatory response to lipopolysaccharide (LPS) in primary cultures of microglia and astrocytes. This effect is achieved by inhibiting pro-inflammatory mediators such as iNOS, IL-6, IL-1β, and TNF-α while simultaneously stimulating the production of anti-inflammatory cytokines such as IL-10. The administration of LPS increased KLF4 expression in both microglia and astrocytes. However, the observed response was attenuated by the application of honokiol. The present study provides further insights into the anti-inflammatory effects of honokiol on central glial cells, thereby substantiating its potential as a therapeutic agent for treating neuroinflammatory conditions [63].

The study conducted by Ye et al. [64] aimed to assess the impact of honokiol on cognitive impairment induced by surgery/anesthesia in mice. The administration of honokiol resulted in the mitigation of surgery-induced cognitive impairment and neuronal cell death, as well as a reduction in neuroinflammation and improvement in OS in the hippocampal region. It was observed that surgery/anesthesia resulted in a significant reduction in the level of expression of SIRT3 in the hippocampus. Conversely, honokiol administration led to a rise in SIRT3 expression, thereby causing a decrease in the acetylation of SOD2. The findings indicate that honokiol-induced activation of SIRT3 may mitigate cognitive decline in mice resulting from surgery and anesthesia. This effect is likely achieved by regulating neuroinflammation and OS in the hippocampus [64].

Magnolol, a structural isomer of honokiol, has been found to protect against MPTP/MPP + -induced toxicity by inhibiting oxidative damage in both in vivo and in vitro models of PD. C57BL/6N mice were administered 30 mg/kg once daily for 4 or 5 days, and both treatments effectively reduced DAT and TH protein levels in the striatum caused by MPTP. However, these interventions did not affect GFAP levels induced by MPTP. Magnolol administration also prevented MPTP-induced lipid peroxidation in the striatum and significantly decreased MPP + -induced cytotoxicity and ROS development in SH-SY5Y cells. These findings suggest that magnolol may confer protection through an antioxidant mechanism in both in vivo and in vitro PD models [65].

A raising body of research has shown that honokiol significantly reduces cognitive deficits brought on by various circumstances [11]. Honokiol is effective in treating newborn discomfort and preventing neurobehavioral problems. Long-term treatment reduces chronic hyperalgesia caused by heat and inhibits acute pain responses 30 min after a formalin assault. Rats with honokiol perform better on learning, memory, and exploratory behavior tests. Treatment normalizes c-Fos expression in the hippocampus and thalamic regions and substance P receptors in the hippocampal alveus. These results suggest honokiol protects newborn rats against acute and long-term pain-induced degradation [66].

### Anxiety and depression

Anxiety disorders are common co-morbidities with neuropsychiatric problems and chronic inflammatory ailments including liver damage. Brain-derived neurotrophic factor (BDNF), OS, and peripheral inflammation are significant factors in their pathogenesis. Around one-eighth of the world's population suffers from anxiety-like syndrome [67]. Anti-anxiety medications currently available on the market cause adverse side effects and have low patient compliance rates. Hence, there is a crucial demand to research and develop safer and more potential novel anti-anxiety therapeutics [68]. To find novel therapeutic agents for treating NDs, research on medicinal plant products is continuously progressing in different countries. In various animal models of psychiatric disorders, some plant species have been shown to have pharmacological effects [69].

Honokiol has been extensively researched on whether it possesses antidepressant and anti-anxiety properties. A study conducted by Sulakhiya et al. [71] found that administering honokiol at a higher dosage resulted in a substantial decrease (*p* < 0.05) of anxiety-like behavior induced by LPS. This was evidenced by an increase in the number of records, the duration spent in the open arm during the EPM test, and an increase in the frequency of entries into the central zone during the OFT. The administration of honokiol prior to lipopolysaccharide exposure reduced peripheral inflammation, as evidenced by decreased levels of plasma IL-1β, IL-6, and TNF-α. Additionally, honokiol treatment led to an improvement in plasma BDNF levels. It prevented liver damage by reducing transaminase levels (ALT and AST), liver OS, and TNF-α activity in mice challenged with LPS.

To summarize, the present study indicates that honokiol positively impacts anxiety-like behavior and liver damage caused by LPS. This effect is likely attributed to the suppression of cytokine production, oxidative stress, and reduction of plasma BDNF level (Table 1). The study's findings indicate that honokiol may have potential as a therapeutic intervention for addressing anxiety and other neurological diseases linked to inflammation and OS [70].

**Table 1:** Summarization of neuropharmacological potential of Honokiol

Diseases	Study model	Dose	Findings	References
Alzheimer’s disease	PS70 cell lines	0.5, 1, 2, 5, 10 and 20 μM	Enhanced SIRT3 expression, reduced Aβ levels	[42]
	Male APPswe/PS1dE9 transgenic mice	20 mg/kg per day, intraperitoneally	Lowered Aβ production and enhanced Aβ phagocytosis by microglia	[43]
	Male wildtype C57BL/6 mice	0.7, 7, and 70 μg/kg	Honokiol exhibited a dose-dependent reduction in hippocampal neural apoptosis, ROS generation, and decline in the membrane potential of mitochondria caused by AβO	[45]
	Male and female PS1V97L-transgenic (Tg) mice	20 mg/kg	Reduced mitochondrial dysfunction and enhancing SIRT3 hyperactivation in the etiology of AD	[46]
	PC12 cells	1 μM, 10 μM, 50 μM, 100 μM	Dose-dependent reduction of ROS, suppression of intracellular Ca elevation, and inhibition of caspase-3 activity	[1]
	Male Sprague–Dawley rats	10^−4^ M,10^−5^ M, 10^−6^ M	Increased extracellular acetylcholine release to 165.5 ± 5.78% of the basal level	[86]
	SAMP8 mice	0.1, 1 mg/kg	Enhanced phosphorylation of Akt	[47]
	Murine microglial cells	5 µM	Increased the expression of PPARγ and PGC1α	[87]
Parkinson's disease	Male NMRI mice	0.1–5 mg/kg, i.p	Improvement of motor dysfunction due to reversal of nigrostriatal dopaminergic neuronal loss	[61]
	Male NMRI mice	5 mg/kg, i.p	Honokiol has been found to exhibit protective and therapeutic properties against motor impairments and dopaminergic progressive damage in mice that have been subjected to 6-OHDA lesioning	[14]
	Male C57BL/6 mice	-	The MPTP model group of mice demonstrated a lower frequency of spontaneous movement than the control group	[62]
	Rat microglia and astrocytes	1-100 µM	The study demonstrates the inhibitory effect of Honokiol on the generation of pro-inflammatory cytokines in primary microglia and astrocytes in response to LPS	[63]
	Adult C57BL/6 mice	10 mg/kg, IP	The administration of Honokiol increased the expression of SIRT3, subsequently leading to a reduction in the acetylation of SOD2	[64]
	Male C57BL/6 N mice	30 mg/kg	Both in vivo and in vitro models of Parkinson's disease show that magnolol's protective actions are mediated through an antioxidative mechanism	[65]
	Neonatal rats	10 mg/kg	Honokiol protects against and lessens the severity of acute and chronic pathological pain in neonatal rats	[66]
Depression and anxiety	Mouse model	10 mg/kg	Improvement of LPS-induced NF-κB activation. reduced the levels of the pro-inflammatory cytokines TNF-α, IL-1βIFN-γ	[88]
	Male mice	20 and 40 mg/kg	Increased 5-HIAA levels. reversed CMS-induced reduction in platelet AC activity via upregulating the cAMP pathway	[89]
	Adult Swiss albino mice	20 mg/kg	Increased brain BDNF levels (*p* < 0.01)	[76]
	Mouse model	2.5, 5, 10 mg/kg	Reduced levels of the enzyme IDO that is involved in the tryptophan pathway, as well as reduced levels of its gene expression	[90]
	Male Wistar rats	2, 4, and 8 mg/kg	Resulted in an upsurge in the amount of GRα (both mRNA and protein) and BDNF (both mRNA and protein)	[74]
	Adult male Swiss albino mice	2.5 and 5 mg/kg; i.p	Improved LPS-induced inflammation	[70]
	SH-SY5Y cells	30 mg/kg	Reduction of the excitotoxicity induced by GLU through the modulation of CB1 receptors	[91]
Cerebral ischemia	Female Wistar albino rats	25 mg	Reduction in ovarian tissue concentrations of malondialdehyde	[82]
	Male Long-Evans rats	10^–6^,10^–7^,10^−8^ g/kg	Reduced the total volume of infarction	[84]
	Male Long–Evans rats	0.01–1.0 μg/kg	Inhibited neutrophil infiltration and ROS production	[80]
	Male S-D rats	0.001–10 mM	Decreased the intensity of nNOS related to PSD95	[81]
	ICR mice and male SD rats	10 and 50 μg/kg	Reduced NO production in the injured brain	[85]
Cerebral cell damage	Sprague–Dawley rat	0.1–10 μM	Reversed mitochondrial dysfunction and cell damage	[2]
	Male Sprague–Dawley rat	1.0 mg/kg	Attenuation of the expression of some of the critical cell cycle proteins such as cyclin D1 and E2F1, degeneration, and apoptosis in the cortex and hippocampus	[92]
	HT22 cells wild-type (AB strain) zebrafish	50 µM 0.15 g/L	Attenuated the levels of ROS, TNF-α, and IL-1β in both the in vivo and in vitro	[93]
	Male Sprague–Dawley rats	20 mg/kg	Decreased the expression of APAF-1 and decreased the apoptosis of neurons	[94]
Spinal cord injury	Male Sprague–Dawley rats	20 mg/kg	Increased chromatin in canali ependymal cells and a decrease in degeneration in multipolar and bipolar neurons	[95]

The centrally depressing effects of honokiol may offer not only the anticonvulsant action of the compound but also the anxiolytic efficacy at low dosages. The GABA_A_ receptor is a common target for anxiolytics like benzodiazepines, which may explain its relaxing effect [10]. Kuribara et al. [71] studied the effects of honokiol on animal behavior, finding that a single oral dose increased exploratory behavior and decreased anxiety-related behavior. Multiple doses, even at a minimal dosage of 0.2 mg/kg, led to fewer motor or cognitive adverse effects. The anxiolytic effect of diazepam was disrupted by both flumazenil and bicuculline. However, combined administration of CCK-4 and caffeine with diazepam led to distinct outcomes. The authors concluded that honokiol has anxiolytic effects with a more favorable adverse effect profile than diazepam. Additionally, their findings suggest that honokiol may operate through alternative processes, as evidenced by their observations with caffeine [71].

One of the goals of recent research was to investigate honokiol's influence on sleeping. It was shown that Honokiol, at doses of 10 and 20 mg/kg, dramatically shortened sleep latency and increased the quantity of NREM sleep, but it had no impact on the duration of REM sleep. This same research team discovered that honokiol stimulated sleep-inducing neurons in the ventrolateral preoptic sections of the brain. The fact that honokiol makes it easier to fall asleep and reduces anxiety points to the possibility of further use for this neolignan in anesthesia [72].

In addition, honokiol treatment can prevent cognitive damage and behavior similar to depression caused by prolonged restraint stress by inhibiting stress on the endoplasmic reticulum (ER) in the hippocampus of rats. In an experiment, groups were given Honokiol (3 and 10 mg/kg) and Imipramine (10 and 30 mg/kg) during the previous week. The MWM test was used to evaluate cognitive function. The group with honokiol showed a significant improvement in cognitive function and behavior, similar to depression. However, imipramine could not stop cognitive damage caused by restraint stress despite suppressing depressive-like behavior [73].

Additionally, higher levels of pro-inflammatory cytokines, GRP78, and CHOP were found in the hippocampus of stressed mice than in those without stress. When administered at 10 mg/kg, honokiol significantly suppressed these cytokines, suggesting that honokiol may positively affect restraint stress. This suggests that honokiol may be an exciting treatment option for neuropathophysiological disorders related to ER stress [73].

A study suggests that honokiol can potentially serve as an antidepressant in rats that have been subjected to chronic unpredictable mild stress (CUMS). This effect is achieved through the regulation of BDNF activity. The administration of honokiol at varying dosages (2, 4, and 8 mg/kg) resulted in the amelioration of behavioral deficits induced by CUMS. The administration of honokiol resulted in normalizing the limbic system hypothalamic–pituitary–adrenal (HPA) axis hyperactivity caused by CUMS, as evidenced by the reduction in serum levels of CRH, ACTH, and CORT. Furthermore, honokiol elicited an upregulation of GRα (both mRNA and protein) and BDNF (both mRNA and protein) within the hippocampal region. The data mentioned above substantiated the antidepressant properties of honokiol, potentially attributed to its capacity to restore the HPA axis function and elevate the BDNF concentration in the hippocampus [74].

The study conducted by Fan et al. [75] demonstrated that the activation of the HIF-1α-VEGF signaling pathway by honokiol resulted in an improvement of depression-like behaviors in rats. Honokiol demonstrated notable antidepressant effects by auguring synaptic plasticity via mechanical means. Honokiol has been observed to trigger the HIF-1α-VEGF signaling pathway both in vitro and in vivo at a molecular level. Additionally, it has been found to enhance the protein expression levels of SYN 1 and PSD 95. The collective findings not only establish an empirical foundation for using honokiol in the clinical management of depression but also propose the possibility of the HIF-1α-VEGF pathway as a viable therapeutic target for depression treatment [75].

The study conducted by Pitta and colleagues revealed that honokiol has the potential to counteract depressive-like behavior and reduce the decline in brain BDNF levels that the administration of corticosterone injections causes over an extended period in mice. The findings indicate that the administration of corticosterone injections for three weeks resulted in a notable increase in the levels of corticosterone in the serum of mice. The administration of corticosterone injections on a repetitive basis resulted in the manifestation of behavior akin to depression in mice. This was evidenced by a marked reduction in sucrose consumption and increased immobility duration during the forced swim test. Additionally, a notable reduction in BDNF levels within the hippocampus was observed in mice treated with corticosterone. The administration of honokiol to mice resulted in a notable reduction in depressive behavior and a concurrent elevation in BDNF levels in mice treated with corticosterone (Table 1) [76].

Honokiol derivatives are expected to be beneficial in anxiety and depressive disorders. *Magnolia *bark extracts have been used to treat anxiety and psychological problems. The primary components of interest consist of biphenyl-type neolignan honokiol and its isomer magnolol. The compound under investigation is a derivative of honokiol, which contains nitrogen (specifically, 3-acetylamino-4′-O-methylhonokiol, abbreviated AMH). This derivative is a very effective modulator of GABA_A_ receptors. This investigation aims to provide a comprehensive understanding of the correlation between the chemical structure and biological activity of nitrogenated biphenyl-neolignan derivatives. This is achieved by analyzing their allosteric regulation and agonistic actions on α1β2γ2S GABA_A_ receptors. The compound 3-acetamido-4′-ethoxy-3′,5-dipropylbiphenyl-2-ol exhibited the most prominent and most robust attachment to IGABA, but the compound 5′-amino-2-ethoxy-3′,5-dipropylbiphenyl-4′-ol shown a much greater potency [77]. Besides, Thirteen novel glucosides of magnolol and honokiol were synthesized by targeted O-glycosylation reactions using two filamentous fungus, *Cunninghamella echinulata* AS 3.3400 and Rhizopus japonicus ZW-4. Minor reactions were reported, including hydroxylation and selective 6″-O-acylation. The findings of the bioassay demonstrated that the combination of glucosides with magnolol and honokiol at a concentration of 10 μM effectively reduced the toxic effects generated by glutamate in SK-N-SH cells, reaching a degree of attenuation similar to that seen with MK-801, a positive control. Nevertheless, there was a noticeable rise in the water solubility of the primary glycosylated compounds [78].

### Cerebral ischemia

Ischemia of the brain, also known as cerebral ischemia, is a frequent cause of acute brain damage and occurs when the blood supply to the brain is restricted. Ischemia of the brain is considered a medical emergency because, if left untreated, it may lead to cerebral infarctions or global hypoxic-ischemic encephalopathy, both of which have the potential to cause death or lifelong impairment [79]. The study results indicate that honokiol was protective against injury caused by focal cerebral ischemia–reperfusion (FCI/R) in rats. Liou et al. [80] reported that the intravenous delivery of honokiol at varying doses (0.01–1.0 μg/kg) either 15 min before (pre-treatment) or 60 min after (post-treatment) middle cerebral artery occlusion resulted in a dose-dependent decrease in the total infarcted volume by 20–70%. Applying honokiol at concentrations of 0.1 and 1.0 μg/kg, either before or after treatment, resulted in a significant reduction in neutrophil infiltration in the brain affected by infarction. According to the findings, honokiol can potentially protect brain tissue from peroxidation of lipids and infiltration of neutrophils in the context of FCI/R injury.

Additionally, it was observed that cerebral infarction resulting from FCI/R is characterized by significant neutrophil infiltration in the affected area during FCI/R [80]. The administration of honokiol microemulsion resulted in a substantial reduction in neural deficit, infarct volume, and brain water level in rats that underwent cerebral ischemia–reperfusion. Additionally, honokiol at concentrations ranging from 0.1 to 10 μM exhibited a significant reduction in damage caused by oxygen-glucose lack or glutamate in fetal rat cortical neurons. Moreover, the outcomes demonstrated that honokiol exhibited a reversible inhibitory effect of approximately 64% on the NMDA current. To summarize, the therapeutic effects of honokiol have been observed to persist for a minimum of 5 h following the beginning of cerebral ischemia. This may be attributed to the disruption of the PSD95-nNOS interaction, reducing neurotoxic nitric oxide (NO) production [81].

In a separate piece of research, the beneficial properties of honokiol on rat models of ischemia and reperfusion damage were studied. In the group that received honokiol, all histological scores, including hemorrhage, cell infiltration, and cellular degradation, were shown to have decreased significantly. Malondialdehyde levels in ovarian tissue were considerably more remarkable in the torsion and detorsion groups than in the sham and honokiol groups. Therefore, it is possible to conclude that honokiol positively impacts ovarian ischemia/reperfusion damage caused by torsion [82].

A study was undertaken to investigate the neuroprotective properties of 002C-3, its possible mechanisms of action in rats, and its initial toxic effects in mice. The present study employed a rat model to assess its impact and underlying mechanism. Additionally, a dose-restricted experiment was conducted to assess the early toxicity of the model. The administration of a single intravenous bolus of 002C-3 at a dose range of 10–50 μg/kg after reperfusion substantially reduced infarct volumes, neurological scores, and brain water contents. When administered at a high effective level of 1000 times the standard dose, 002C-3 did not exhibit any observable toxicity in mice [83].

Additionally, the mean weight of mice treated with 002C-3 was comparable to that of the control group that received the vehicle. Conversely, magnolol was found to cause significant toxicity and mortality in the mice. To summarize, the compound 002C-3 exhibits a noteworthy safeguarding impact against cerebral ischemia–reperfusion injury, surpassing the potency of magnolol. This beneficial effect may be attributed to its ability to prevent autophagy and apoptosis. Additionally, 002C-3 displays reduced toxicity in comparison to magnolol. The above findings gave valuable information for subsequent investigation and advancement [83].

The neuroprotective potential of honokiol was investigated by Liou et al. [84] in rats that underwent focal cerebral ischemia. The use of honokiol was carried out via IV in two distinct groups: the pre-treatment groups, where the administration took place for 15 min before the obstruction of the right middle cerebral artery, and the treatment groups, where the administration occurred after removing both common carotid artery clips. The study findings indicate that an IV infusion of honokiol at doses of 10^–8^, 10^–7^, and 10^–6^ g/kg did not result in significant hemodynamic alterations in either of the groups. Honokiol exhibited a substantial decrease in the overall infarction volume in both treatment and pre-treatment groups at doses of 10^–7^ and 10^–6^ g/kg. The findings of this investigation propose that honokiol exhibits significant potential as a neuroprotective agent in cases of focal cerebral ischemia. The positive impact could be attributed to the antioxidant [84].

Brain ischemia–reperfusion damage may be prevented in rats by administering a synthetic analog of honokiol named W007B. This compound preserves brain tissue by reducing inflammation, autophagy, and apoptosis. Bu et al. [85]’s experimental results indicate that the administration of w007B (at doses of 10 and 50 μg/kg, intravenously immediately after reperfusion) resulted in a substantial reduction in neurological deficit scores, a decrease in infarct size, and a reduction in brain water content. Furthermore, the administration of 50 μg/kg w007B within 3 h after reperfusion (5 h after ischemia) significantly mitigated ischemia-induced brain injury. Furthermore, there was no indication of any toxic effects upon administering a solitary dose of 50 mg/kg w007B (1000 times greater than the maximum effective dose, IP). The study reported a notable decrease in caspase-3 activity and NO production in the injured semi-brain upon administration of w007B.

Additionally, the nuclear level of the p65 subunit of NF-κB was observed to be reduced. Moreover, it elicited a decrease in the expression of Beclin-1 and LC3B-II while inducing an elevation in the level of p62, which are proteins associated with autophagy, in the hemisphere subjected to ischemia/reperfusion injury. In summary, it has been observed that w007B demonstrates a neuroprotective impact on cerebral ischemia–reperfusion injury, with a broader therapeutic time frame and enhanced safety. This effect’s mechanisms may be linked to its anti-inflammatory, anti-apoptotic, and anti-autophagic properties. The findings indicate that w007B exhibits considerable promise as a clinical neuroprotective agent for managing ischemic stroke [85].

### Spinal cord injury

Spinal cord injuries (SCIs) are severe medical conditions that have long-lasting effects on patients' lives. Primary and secondary stages of illness develop after SCI. When the spinal cord is compressed, neurons and glia are injured, and axons are lost. Because of the activation of molecular cascades and messenger routes, secondary damage is triggered when axons are severed, or cell membranes are ruptured—secondary damage results from the death of neurons and glial cells from free radicals and OS [96]. Damage to the spinal cord's myelin sheath can also cause its breakdown and demyelination.

The inflammation itself partly causes secondary damage to the spinal cord caused by inflammation. Because of its potent antioxidant and anti-inflammatory characteristics, honokiol is a neuroprotective compound in certain studies. The honokiol compound has an anti-inflammatory effect, and a recently discovered essential target for this activity is Klf4. It has not yet been determined whether or not honokiol can suppress the inflammatory response in a rat model of SCI by regulating the expression of Klf4. According to the findings of Liu and colleagues, honokiol lowers the severity of histopathology, attenuates inflammation, and downregulates the expression of Klf4 in rats with spinal cord injuries. In addition, honokiol enhanced the functional outcome after traumatic SCI and decreased the amount of histopathology that was present. By inhibiting the body's inflammatory response, honokiol not only helps patients recover from spinal cord injuries more quickly and with less pain, but it also minimizes the amount of secondary damage to the tissues that occurs as a result of the injury [97].

A separate study has indicated that honokiol demonstrates protective properties towards neural myelin sheaths in the aftermath of compressed SCI. This is achieved by impeding oligodendrocyte apoptosis through controlling ER-mitochondrial interactions. Following a six-day intervention with honokiol, notable improvements were observed in the locomotor function and pathomorphological alterations of the myelin sheath in rats with chronic spinal cord dorsal column damage (CSCD) compared to the control group. These improvements were accompanied by a significant reduction in the levels of active caspase-3, caspase-12, and cytochrome C. Honokiol has been found to potentially enhance locomotor function and safeguard the neural myelin sheath against demyelination by inhibiting apoptosis of oligodendrocytes (OLs) through the mediation of the ER-mitochondria pathway after cervical SCI [12].

Biochemical and immunohistochemistry research was conducted by Firidin et al. [95] to study the preventive impact of the antioxidant honokiol on neuronal and glial cells in the spinal cord following a lesion to the spinal cord. As a result of the trauma, there was a rise in the production of caspase-3 in multipolar cells and a positive caspase-3 response in solitary glial cells. *Canalis ependymalis* cells and many glial cells within the honokiol group were found to have low levels of caspase-3 activation. Following a traumatic lesion to the spinal cord, alterations, including degeneration and apoptosis in neuron and glial cells, as well as enhanced inflammation and thrombosis in blood vessels, emerged. The damage was caused by lipid peroxidation as a direct result of an increase in OS. After the honokiol treatment, there was a significant decrease in OS, inflammation, and angiogenetic impact in blood vessels. It is believed that honokiol has a beneficial impact on triggering apoptotic signals in neuronal and glial cells in situations where there has been damage [95].

## Pharmacokinetic profile

Pharmacokinetic studies on honokiol medication should be conducted, as well as more precise dose designs for diverse conditions and new drug delivery systems to improve bioavailability in hospital settings. Honokiol primarily undergoes hepatic metabolism and in vivo biotransformation, wherein glucuronidation and sulfation serve as the principal metabolic pathways for the conversion of honokiol into mono-glucuronide honokiol and sulfated mono-hydroxy honokiol before its elimination [98, 99]. Honokiol has a biphasic kinetic profile characterized by an initial rapid distribution phase and a subsequent slower elimination phase. The elimination half-life for honokiol after intravenous administration is 49.2 min for a 5 mg dose and 56.2 min for a 10 mg dose [100, 101]. Plasma concentrations of honokiol in mice treated with liposomal honokiol remained above 30 and 10 μg/mL for 24 and 48 h, respectively.

In contrast, mice treated with free honokiol experienced a rapid decrease in plasma concentration (< 5 μg/mL) within 12 h. These results suggest that liposomal honokiol extends the duration of honokiol circulation in the blood of mice with A549 xenograft tumors, compared to free honokiol [102, 103]. The oral administration of Honokiol to rats resulted in rapid distribution in all organs, with the highest concentration accumulation in the brain, liver, and kidneys [104, 105]. A maximal plasma honokiol concentration of about 1100 µg/mL was achieved in nude mice when honokiol was injected intraperitoneally at a dose of 250 mg/kg. This maximal plasma honokiol concentration was achieved at 27.179 ± 6.252 min after administration. According to the plasma pharmacokinetic curve, the half-life of absorption was 10.121 ± 2.761 min, whereas the elimination half-life was 5.218 ± 0.461 h [106, 107]. The low oral bioavailability of honokiol and magnolol can be due to their substantial first-pass metabolism, low water solubility, and poor absorption [108, 109]. The distribution of magnolol is primarily observed in the kidney, lung, liver, brain, and heart. The liver exhibits the most substantial concentrations of magnolol-glucuronides and magnolol [110, 111].

## Clinical status

Implementing clinical trials to establish evidence for improving the management of health-related concerns is necessary. A clinical trial could evaluate strategies to enhance patient compliance with therapeutic regimens [112]. Although several in vivo and in vitro tests on the neuropharmacological activities of honokiol have been conducted, only a few clinical trials have been conducted.

The use of *Magnolia *bark extract (MBE) as a potential source of beneficial components has been observed in various human research investigations, either in isolation or in combination with other herbal components. In a clinical trial evaluating the efficacy of a dietary supplement containing MBE and phellodendron compared to a placebo, participants were administered a daily dosage of 11.25 mg of honokiol and 0.75 mg of berberine [113]. Forty-two overweight women volunteered for the trial; three dropped out due to side effects (two in the experimental group and one in the placebo arm). Heartburn, shaky hands, prebilabial numbness, sexual dysfunction, thyroid malfunction, weariness, and headaches were some of the symptoms mentioned by the two research participants. No substantial treatment-related adverse events were reported throughout the research, and no statistically significant variations in biochemical markers were seen between the groups [113, 114].

## Toxicological profile

The potential risks associated with honokiol cannot be disregarded entirely, as its scientific implementation to people at therapeutic levels has been limited, thereby restricting the assessment of its adverse effect profile. When administered excessively, possible hazards can be anticipated, such as heightened hemorrhaging and probable neurological damage [10]. An investigation was conducted to determine the honokiol microemulsion's acute and subchronic toxicities. During the acute toxicology testing, the mice received graded dosages of honokiol microemulsion through an IV, and they were monitored every day for 14 days for signs of toxic manifestations and death. In the research on sub-chronic toxicity, rats were given honokiol microemulsion injections at 100, 500, and 2500 g/kg body weight (BW) over a month. Following a treatment period of 30 days and a recovery period of 14 days, the rats were slaughtered for hematological, biochemical, and histological analysis. According to the results of acute toxicology tests, the predicted median lethal dose (LD50) in mice was 50.5 mg/kg of body weight. In the subchronic toxicity studies, the amount that produced a non-toxic response was 500 µg/kg b.w. According to the results of this research, the honokiol microemulsion is harmless up to 500 g/kg body weight; nevertheless, it does cause irritation to the vascular tissue at the location of injection, which should be considered when developing therapeutic medications [115]. The present investigation assessed the embryonic and fetal developmental effects of honokiol microemulsion. During the gestational period from day 6 to day 15, pregnant SD rats were subjected to intravenous injection of the drug at varying dose levels of 0, 200, 600, and 2000 g/kg/day. The gravid animals were monitored for variations in body mass and any anomalous modifications and were subsequently undergoing cesarean delivery on gestational day (GD) 20. All offspring procured from the cesarean delivery were evaluated through external inspection and visceral and skeletal examinations. The groups administered with honokiol microemulsion did not exhibit any external modifications or malformations in the visceral and skeletal systems that could be attributed to the treatment. Nonetheless, embryonic-fetal developmental toxicity was detected at a dose level of 2000 g/kg/day, which reduced the body and tail length of fetuses. The established no-observed-adverse-effect level (NOAEL) for honokiol microemulsion is 600 g/kg/day [116].

Honokiol is identified as an effective inhibitor of artery thrombosis. Therefore, it is recommended to use caution when administering honokiol to patients with coagulopathy or those at risk of bleeding or hemorrhage [17]. The categories mentioned above of patients comprise individuals with hemorrhagic stroke, those exhibiting hemorrhagic alterations after ischemic stroke, patients undergoing coumadin or therapeutic Lovenox treatment, and individuals with clotting conditions such as hemophilia or von Willebrand's deficiency. Honokiol exhibits neuroprotective properties at lower doses; however, it is also shown to induce neuronal degeneration in vitro at elevated doses (specifically, 100 µM) [10, 117].

Cosmetics with *M. officinalis* are currently being promoted as anti-aging products. Like other extracts of *Magnolia*, MBE has also been associated with cases of allergic contact dermatitis, indicating that this component may be an uncommon allergen [118].

The utilization of *Magnolia* bark extract (MBE) as a helpful component has been observed in various human research investigations, either in isolation or in combination with other herbal components. In a clinical trial evaluating the efficacy of a dietary supplement containing MBE and phellodendron compared to a placebo, participants were administered a daily dosage of 11.25 mg of honokiol and 0.75 mg of berberine [113]. Forty-two overweight women volunteered for the trial; three dropped out due to side effects (two in the experimental group and one in the placebo arm). Heartburn, shaky hands, prebilabial numbness, sexual dysfunction, thyroid malfunction, weariness, and headaches were some of the symptoms mentioned by the two research participants. No substantial treatment-related adverse events were reported throughout the research, and no statistically significant variations in biochemical markers were seen between the groups [113, 114].

Both magnolol and honokiol were tested for their possible mutagenic and genotoxic effects, although only MBE was used, not the pure chemicals themselves. Human fetal small intestine epithelial noncancerous cells, often employed to examine the biological implications of food or medication, were employed in recent research to assess the genotoxic effects of an aqueous extract of *M. officinalis*. Thin-layer chromatography was employed to scrutinize the MBE following the European Pharmacopoeia 8.4 monographs. The analysis results revealed that magnolol and honokiol were consistent with the description provided in the European Pharmacopoeia. The decoction contained only negligible amounts of these two neolignans [119].

There may be pharmacokinetic drug interactions between honokiol and drugs that are co-administered and metabolized by CYP2C19, CYP2C8, CYP1A2, CYP2C9, and UGT1A9 [120]. Honokiol is a poor CYP2B6 inducer. Hence, it is unlikely to accelerate the metabolism of other CYP2B6 substrates and create pharmacokinetic drug interactions in human subjects [121]. The prolonging of propofol anesthesia was shown in rats when magnolol inhibited glucuronidation. However, pigs and monkeys, which have metabolic similarities to humans, had a lower sensitivity to glucuronosyltransferase inhibition [114, 122]. A study revealed that pharmacokinetic interactions occur between Magnolol and Piperine in rats. The pharmacokinetic properties of piperine remained unchanged when administered concurrently with magnolol at a 20 mg/kg dose. However, at a high dosage of 40 mg/kg, the maximum concentration of piperine dropped significantly from 4.30 ± 1.47 µg/mL to 2.50 ± 0.78 µg/mL (*p* < 0.05). No significant differences were observed in the area under the curve (AUC) and half-life (t1/2) of piperine in the plasma when given alone or in combination with magnolol [123].

## Concluding remarks and future directions

Even while technological advances have significantly sped up the process of assessing phytochemicals, there is still a lot of work to be done until we have a more definite understanding of the neurotherapeutic advantages of herbal medications. As a consequence of our findings and other studies' findings, we have concluded that honokiol's ability to change several signaling pathways makes it potentially useful as a medicine for treating NDs. Despite the limitations of the ongoing clinical study, honokiol is showing great potential as a potential treatment for a wide range of NDs, including AD, PD, cerebral ischemia, anxiety, depression, spinal cord, and others. Even though a significant amount of preclinical research studies have been carried out to evaluate the neurotherapeutic potential of honokiol, the compound's clinical potential has not yet been used. There is a need for more research to improve the bioavailability of honokiol and for clinical studies to be carried out to evaluate the therapeutic efficacy of these compounds.

## Data Availability

The datasets used and/or analyzed during the current study are available from the corresponding author upon reasonable request.
